# Hypophosphatemia as Unusual Cause of ARDS in Cushing’s Syndrome Secondary to Ectopic CRH Production. A Case Report

**DOI:** 10.1100/tsw.2008.20

**Published:** 2008-02-06

**Authors:** Stefania Mondello, Vincenzo Fodale, Salvatore Cannav, Carmela Aloisi, Barbara Almoto, Michele Buemi, Letterio B. Santamaria

**Affiliations:** ^1^ Department of Internal Medicine, University of Messina, School of Medicine, Policlinico Universitario “G.Martino”, 98125 Messina, Italy; ^2^ Department of Neurosciences, Psychiatric and Anesthesiological Sciences, University of Messina, School of Medicine, Policlinico Universitario “G.Martino”, 98125 Messina, Italy

**Keywords:** acute respiratory failure, ARDS, hypophosphatemia, Cushing's syndrome, ectopic ACTH production

## Abstract

Hypophosphatemia is an unusual cause of acute respiratory distress syndrome (ARDS). We describe a hypophosphatemia-related ARDS case report of a 50-year-old woman with ACTH dependent Cushing's syndrome secondary to ectopic CRH production. The patient clinically showed hypotension tachypnea and increasing dyspnea. Laboratory data showed carbohydrate intolerance, severe hypokalemia, and hypophosphatemia. Arterial blood gases measurement revealed hypocapnia and elevation in bicarbonate values. Chest X-ray showed diffuse bilateral alveolar infiltrates similar to acute pulmonary edema and Kerley's striae. Chest CT scan evidenced diffuse ground glass opacification, bilateral patchy consolidation, and fibrosis, compatible with the recovery phase of ARDS. Clinical symptoms and laboratory examinations supported the diagnosis of ARDS. The patient was managed with supplemental potassium, octreotide, and oxygen therapy. Hypophosphatemia was managed by treating the underlying disorder. Successive surgical removal of the adrenal gland led to complete resolution of Cushing's syndrome. In conclusion, although rare and associated with specific risk factors, hypophosphatemia should be suspected in patients who develop unexplained ARDS.

Hypophosphatemia is a rare condition, occurring in 1–5% of all hospitalized patients[1,2,3]. The most frequent risk factors are diabetic ketoacidosis, chronic obstructive pulmonary disease (COPD) and asthma, malignancy, long-term treatment with total parenteral nutrition, and alcoholism[4,5,6,7].

In patients with ectopic ACTH-induced Cushing's syndrome, hypophosphatemia is related to both metabolic alkalosis and glucocorticoid excessive related activity, inducing phosphaturia and intracellular shift of phosphorus, because of the stimulation glycolysis and formation of phosphorylated glucose compounds. Phosphate, involved in the glycolitic pathway and the generation of the high-energy compounds[1,2], also affects muscle[8] and diaphragmatic contractility[2].

Hypophosphatemia is reported as an unusual cause of acute respiratory distress syndrome (ARDS)[4], but there are no available reports of ARDS related to hypophosphatemia because of ectopic CRH-induced Cushing's syndrome.

## CASE REPORT

A 50-year-old woman presented with a history of cephalea, polyarthritis, and fever. Erysipela was diagnosed. The patient was treated with antibiotic therapy, diuretics, and corticosteroids, but without effect on her clinical condition. In the same period, there was elevation of glucose values consistent with diabetes mellitus.

One month later, the patient was admitted to a medical center because of severe hypokalemia ($K^+$ <2.5 mEq/l) and metabolic alkalosis. Hypokalemia was resistant to supplemental potassium administration. Several days after admission, she developed acute respiratory failure related to ARDS, with diffuse bilateral pulmonary infiltration. There was arterial hypertension (170/100 mmHg).

As her electrolytic, metabolic, and respiratory conditions had worsened, the patient was admitted to the metabolic intensive care unit. Physical examination revealed obesity (weight 86 kg, height 158 cm), skin modifications including epidermal atrophy, abdominal purple striae, hyperpigmentation, hypertrichosis, amyotrophy, and muscle hypotony. The patient was tachypnoic (respiratory rate 22 breaths/min) and dyspneic. An electrocardiogram showed normal cardiac activity, although there was severe hypokalemia. Arterial blood gases measurement evidenced alkalosis: pH 7.5, $pCO_2$ 43.8 mmHg, $pO_2$ 51.8, $HCO_3$ 33.5 mmol/l. Blood cultures were negative. Laboratory values on hospital and metabolic intensive care unit admissions are listed in Table 1, while endocrine values are listed in Table 2.

The patient refused the invasive procedure to establish Pulmonar Capillary Pressure value. However, an echocardiogram rejected cardiac failure.

Serum chromogranin A values were >1,000 ng/ml (normal values <100). Increased serum cortisol (135 µg/dl), ACTH (332 pg/dl) levels, and urine free cortisol excretion rates (16.270 µg/24 h) were found. These results supported the diagnosis of possible ectopic ACTH secretion. Abdominal CT scans showed a 9.55- × 6.39-cm mass attached to the right adrenal gland (Fig. 1). A total body 111 In-octreotide scan was negative, but the administration of octreotide (100µg s.c.) induced the prompt decrease of ACTH and cortisol levels. Brain MRI revealed a partially empty pituitary fossa, without modifications in the adenopituitary gland. Neurological examination showed hypotonic muscle with signs of weakness and atrophy. Deep tendon reflexes were absent. Paresthesias and sensory deficit were present in the lower limbs.

Chest X-ray showed diffused bilateral alveolar infiltrates similar to acute pulmonary edema and Kerley's striae (Fig. 2). After elimination of all other possible respiratory, cardiac, or infectious causes, hypophosphatemia (serum value <1.1 mg/dl) was therefore suggested as the possible cause of acute respiratory distress.

The patient was treated with octreotide (0.1 mg, subcutaneously, three times daily), which induced a significant regression of clinical signs of hypercortisolism, normalization of serum cortisol levels, and urinary-free cortisol within 2 weeks. To achieve a potassium level of 3.5 mmol/l, the patient required oral (potassium chloride, 600 mg, two times daily) and intravenous (potassium chloride, 80 mEq/die) potassium administration. Respiratory failure was treated with oxygen therapy. An $F_IO_2$ 32% was given via Venturi's mask (16–18 h daily) under repeated arterial blood gases monitoring.

Twenty days after the beginning of therapy a chest CT scan showed diffused ground glass opacification, bilateral patchy consolidation, and fibrosis, compatible with recovery phase of ARDS, and bilateral pleural effusion (Fig. 3).

Thirty days after admission, the patient's clinical conditions improved. She underwent surgical right adrenalectomy. Histological examination and immuno- and histochemical characterization revealed findings consistent with neuroendocrine tumor. Immunoreactive CRH was found in almost all resected tissue.

Immunoreactive growth hormone, parathyroid hormone, or gastrin was not found in the same tumour tissue.

**TABLE 1**
**Laboratory Values**

| | Hospital Admission | Metabolic ICU Admission |
|---|---|---|
| Arterial blood pH | 7.53 | 7.59 |
| Arterial blood pO2 (mmHg) | 67 | 51.8 |
| Arterial blood pCO2 (mmHg) | 34.3 | 43.8 |
| HCO3- (mEq/l) | 29.5 | 33.5 |
| Red blood cell count (cells/mmc) | 3.040.000 | 2.190.000 |
| Ht % | 27 | 20 |
| Hb (gr/dl) | 8.9 | 6.8 |
| Reticulocytes % | — | 9 |
| White blood cell count (cells/mmc) | 8.200 | 3.600 |
| Platelet count (cells/mmc) | 178.000 | 152.000 |
| Glucose (mg/dl) | 271 | 250 |
| Sodium (mmol/l) | 139 | 134 |
| Potassium (mmol/l) | 1.9 | 1.5 |
| Chloride (mEq/l) | 102 | 100 |
| Calcium (mg/dl) | 7.2 | 7.4 |
| Phosphorus (mg/dl) | 1.4 | 1.1 |
| Magnesium (mg/dl) | 1.2 | 1.2 |
| Creatinine (mg/dl) | 0.30 | 0.30 |
| Urea nitrogen (mg/dl) | 14 | 25 |
| LDH (mg/dl) | 1174 | 980 |
| CPK (IU/l) | 206 | 41 |
| Serum total bilirubin (mg/dl) | 1.68 | 1.8 |
| Unconjugated bilirubin (mg/dl) | 1.20 | 1.4 |
| Intracellular potassium (mEq/l) | — | — |
| Serum osmolality (mOsm/kg) | 276 | 282 |
| Ionized calcium (mmol/l) | — | 0.70 (n.r. 1.10–1.30) |

**TABLE 2**
**Endocrine Laboratory Values**

| | Values | Reference Range |
|---|---|---|
| AM cortisol (μg/dl) | 135 | 5–25 |
| ACTH (pg/ml) | 332 | 0–50 |
| Urinary-free cortisol (μg/24 h) | 16.270 | <50 |
| Serum aldosterone (ng/dl) | 28.10 | 10–160 |
| DHEAS (μg/dl) | 35.7 | 35–430 |
| 17OHPg (ng/ml) | 7.1 | 0.23–1.36 |

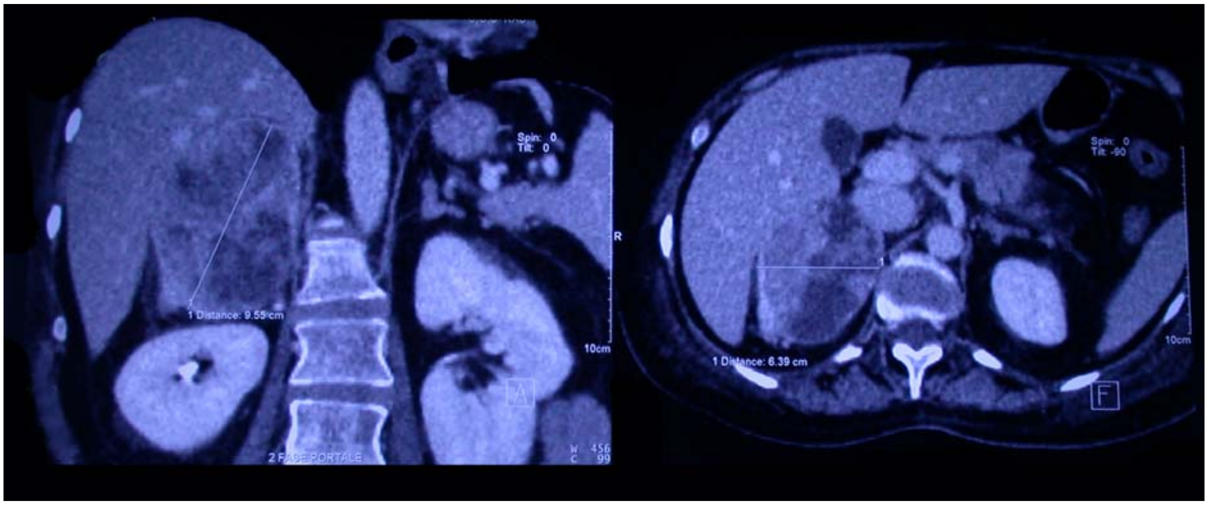

**FIGURE 1.** CT scan showing a 9.55- × 6.39-cm round mass attached to the right adrenal gland.

The postoperative course was uneventful, and the patient did not need hydrocortisone replacement therapy. All clinical and biological signs of Cushing's syndrome were completely resolved.

## DISCUSSION

Ectopic CRH-induced Cushing's syndrome is one of the most rare paraneoplastic syndromes. Patients show clinical manifestations associated with classic Cushing's syndrome: muscle weakness, swelling, hyperpigmentation, hypertension, carbohydrate intolerance, severe and treatment-resistant hypokalemia, and metabolic alkalosis[9,10,11,12,13]. Normal glucocorticoid mineral activity is increased, with decreasing renal absorption of calcium and hypocalcemia. Decrease in plasma calcium increases parathyroid hormone (PTH) gene expression. PTH, with glucocorticoids, decreases renal $PO_4$ reabsorption mainly in the proximal tubule, inducing phosphaturia, and also decreases plasma phosphate[14,15,16]. Moreover, excessive glucocorticoid secretion leads to hyperglycemia and excessive insulin secretion, stimulating glycolysis and leading to the formation of phosphorylated glucose compounds and intracellular shift of phosphorus, the major cause of hypophosphatemia[15,17]. Hypophosphatemia can also be due to alkalosis; in fact, increased pH stimulates glycolysis[7,15,18].

Severe hypophosphatemia should be considered a marker of poor outcome for disorders of the skeletal muscle, cardiac, respiratory, hematological, and central nervous systems[5]. Myalgias, muscle weakness, and anorexia, usually the earliest symptoms, typically occur at serum levels below 1.5 mg/dl. At phosphate levels below 0.8 mg/dl, neurological symptoms (paresthesias, tremor, confusion, decreased deep tendon reflexes, and coma) may progressively develop[7,8]. In accordance, our patient described in the current report showed an abnormal neurological examination.

Rhabdomiyolysis may occur due to structural and functional disturbances in skeletal muscle membranes[8,18]. In fact, our patient showed abnormal CPK and LDH values. Other serious clinical disorders resulting from hypophosphatemia include congestive heart failure related to reduced cardiac contractility and respiratory failure secondary to diaphragmatic weakness[3,8,17,19,20].

The effects of hypophosphatemia on diaphragmatic function are also well known[21]. Intravenous administration of phosphate has been shown to improve diaphragmatic function in mechanically ventilated patients with hypophosphatemia and acute respiratory failure[22]. The increase in contractile properties of the diaphragm (about 50%), correlated with the increase in trans-diaphragmatic pressure, was found after correction of hypophosphatemia[4]. These results strongly suggest that hypophosphatemia impairs the contractile properties of the diaphragm.

Different mechanisms were correlated to impairment of muscle contractility. Hypophosphatemia is associated with low intracellular ATP levels, which may form the basis of the observed muscle weakness[23,24]. Acute phosphate depletion may be associated with membrane dysfunction, leading to high intracellular water, high intracellular potassium, and low intracellular sodium, which also leads to contractile dysfunction[25].

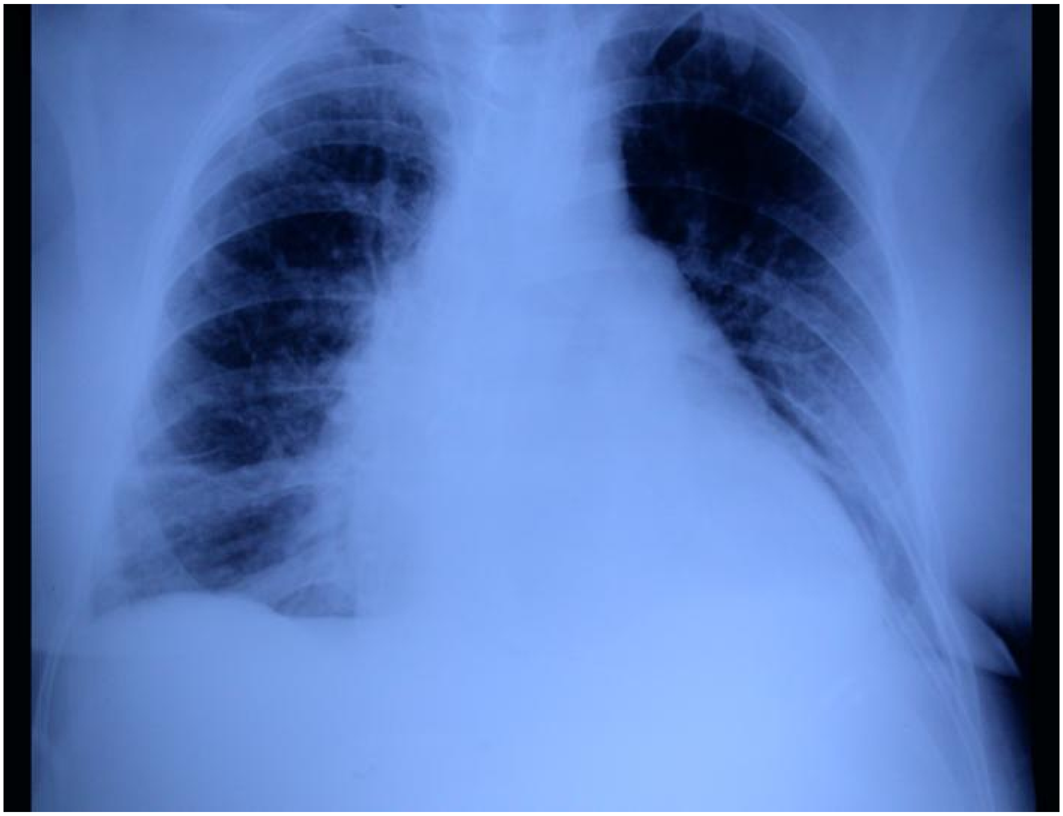

**FIGURE 2.** X-ray image showing diffuse bilateral alveolar infiltrates and Kerley's striae.

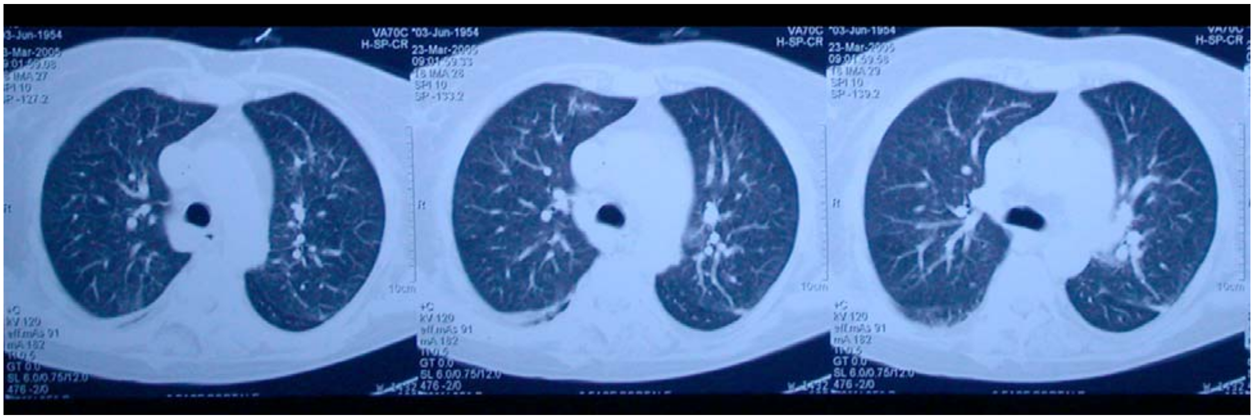

**FIGURE 3.** CT image of thorax showing diffuse ground glass opacification, bilateral patchy consolidation.

Serum phosphorus levels are indicators of muscle cell phosphorus stores. Intracellular phosphate deficit causes energy deficit, as it is involved in the glycolitic pathway and the generation of the high-energy compounds adenosine monophosphate (AMP), adenosine triphosphate (ATP), and phosphocreatine, the body's main reservoirs of biochemical energy[7,8,26]. These same mechanisms are involved in neurological symptoms.

In addition, erythrocyte concentrations of 2,3-diphosphoglycerate decrease, enhancing the affinity of hemoglobin for oxygen and reducing oxygen release from tissue[3,4,14].

The patient's persistent anemia was, most likely, due to hypophosphatemia. Hypophosphatemia can be the cause of hemolysis due to increased erythrocyte membrane rigidity[3,7,8,19]. Phosphate is a vital component of the lipid bilayer of cell membranes, mostly in the form of the phospholipids phosphatidylcoline, phosphatidylserine, phosphatil-dylethanol-amine, and sphingomyelin[27].

Our patient presented acute respiratory failure with features that met the consensus criteria for ARDS: bilateral infiltrates on chest radiography, severe hypoxemia (defined by a ratio of the partial pressure of arterial oxygen to the fraction of inspired oxygen that is ≤200), and no sign of left atrial hypertension[28]. Although ARDS is most often seen in association with infections and trauma, it can result from numerous uncommon causes that may necessitate specific management strategies. One such situation is ARDS in patients with hypophosphatemia.

The patient was treated with octreotide because the acute administration of this drug decreased ACTH and cortisol levels. Also ketoconazole can be used to normalize adverse function. In one series, it decreased both cortisol and ACTH production, and normalized serum cortisol levels in 38% of the patients with ectopic ACTH secretion[29,30). In addition, ketoconazole may help to prevent ARDS by suppressing the formation and release of tumor necrosis factor from macrophages, and this was desirable in the light of respiratory failure[31]. Moreover, several studies indicate that ketoconazole may play a role in ARDS. Ketoconazole inhibition of alveolar macrophage proinflammatory mediators may be of benefit in preventing ARDS by minimizing neutrophil infiltration and microvascular thrombosis. Inhibition of 5-lipoxygenase and thromboxane synthetase, without affecting cyclooxygenase, may offer a selective advantage by allowing production of other homeostatic eicosanoids[32].

In conclusion, in the case report here presented, clinical symptoms and laboratory examinations supported the diagnosis of ARDS. Differential diagnosis was performed and hypophosphatemia was therefore suggested as a possible cause of ARDS. Although rare and associated to specific risk factors, hypophosphatemia should be suspected as a possible cause in patients who develop unexplained ARDS.

## References

1. Baran, D.T. (1991) Disorders of mineral metabolism. In *Intensive Care Medicine*. 2nd ed. Rippe, J.M., Ed. Little Brown, Boston. pp. 987–994.
2. Rutecki, G.W. and Whittier, F.C. (1997) Life-threatening phosphate imbalance: when to suspect, how to treat. *J. Crit. Illness* **12**, 699–704.
3. Fiaccadori, E., Coffrini, E., Ronda, N., et al. (1990) Hypophosphatemia in course of chronic obstructive pulmonary disease. *Chest* **97**, 857–868.
4. Le Corre, A., Veber, B., and Dureuil, B.(2000) An unusual cause of acute respiratory distress. *Ann. Fr. Anesth. Reanim*. **19**, 549–551.
5. Knochel, J.P. (1977) The pathophysiology and clinical characteristics of severe hypophosphatemia. *Arch. Intern. Med*. **137**, 203–220.
6. Knochel, J.P. (1977) Hypophosphatemia. *Clin. Nephrol*. **7**, 131–137.
7. Shiber, J.R. and Mattu, A. (2002) Serum phosphate abnormalities in the emergency department. *J. Emerg. Med*. **23**, 395–400.
8. Subramanian, R. and Khardori, R. (2000) Severe hypophosphatemia: pathophysiologic implications, clinical presentation, and treatment. *Medicine* **79**, 1–8.
9. Wajchenberg, B.L., Mendonca, B., Liberman, B., et al. (1995) Ectopic ACTH syndrome. *J. Steroid Biochem. Mol. Biol*. **53**, 139–151.
10. Carey, R.M., Varma, S.K., Drake, C.R., Jr., et al. (1984) Ectopic secretion of corticotropin-releasing factor as a cause of Cushing's syndrome. A clinical, morphologic, and biochemical study. *N. Engl. J. Med*. **311**, 13–20.
11. Orth, D. (1995) Cushing's syndrome. *N. Engl. J. Med*. **332**, 791–803.
12. Newell-Price, J., Trainer, P., Besser, M., et al. (1998) The diagnosis and differential diagnosis of Cushing's syndrome and pseudo-Cushing's states. *Endocr. Rev*. **19**, 647–672.
13. Wajchenberg, B.L., Mendonca, B.B., Liberman, B., et al. (1994) Ectopic adrenocorticotropic hormone syndrome. *Endocr. Rev*. **15,** 752–787.
14. Rose, B.D. (1994) *Clinical Physiology of Acid-Base and Electrolyte Disorders.* 4th ed. McGraw-Hill, New York.
15. Weisinger, J.R. and Bellorin-Font, E. (1998) Magnesium and phosphorus. *Lancet* **352**, 391–396.
16. Bellorin-Font, E., Milanes, C.L., and Urbina, D. (1990) The regulation of sodium phosphate cotransport in the kidney. In *Diuretics*. Vol. 2. Puschett, J. and Greenberg. A., Eds. Elsevier Science, London. pp. 427–433.
17. Brautbar, N., Leibovici, H., and Massry, S.G. (1983) On the mechanism of hypophosphatemia during acute hyperventilation: evidence for increased muscle glycolysis. *Miner. Electrolyte Metab*. **9**, 45–50.
18. Marik, P.E. and Bedigian, M.K. (1996) Refeeding hypophosphatemia in critically ill patients in an intensive care unit. A prospective study. *Arch. Surg*. **131**, 1043–1047.
19. Vanatta, J.B., Whang, R., and Papper, S. (1981) Efficacy of intravenous phosphorous therapy in the severely hypophosphatemic patient. *Arch. Intern. Med*. **141**, 885–887.
20. Knochel, J.P. (1985) The clinical status of hypophosphatemia: an update. *N. Engl. J. Med.* **313**, 447–449.
21. Newman, J.H., Neff, T.A., and Ziporin, P. (1977) Acute respiratory failure associated with hypophosphatemia. *N. Engl. J. Med*. **296**, 1101–1103.
22. Aubier, M., Murciano, D., Lecocguic, Y., et al. (1985) Effect of hypophosphatemia on diaphragmatic contractility in patients with acute respiratory failure. *N. Engl. J. Med*. **313**, 420–424.
23. Lichtman, M. A., Miller, D.R., Cohen, J., et al. (1971) Reduced red cell glycolysis 2,3-diphosphoglycerate, adenosine triphosphate concentration, and increased hemoglobin-oxygen affinity caused by hypophosphatemia. *Ann. Intern. Med.* **74,** 562–568.
24. Lichtman, M.A., Miller, D.R., and Freeman, R.B. (1969) Erythrocyte adenosine triphosphate depletion during hypophosphatemia in a uremic subject. *N. Engl. J. Med.* **280**, 240–244.
25. Montanari, A., Borghi L., Curti, A., et al. (1984) Skeletal muscle abnormalities in acute hypophosphatemia during total parenteral nutrition. *Miner. Electrolyte Metab.* **10,** 52–57.
26. Polancic, J.E. (2000) Principles, procedures, correlations. In *Clinical Chemistry*. 4th ed. Bishop, M.L., Ed. Lippincott Williams & Wilkins, Philadelphia. pp. 314–316.
27. Berne, R.M. and Levy, M.N. (1988) *Physiology*. 2nd ed. CV Mosby, St.Louis.
28. Bernard, G.R., Artigas, A., Brigham, K.L., et al. (1994) The American-European Consensus Conference on ARDS. Definitions, mechanisms, relevant outcomes, and clinical trial coordination. *Am. J. Respir. Crit. Care. Med*. **149**, 818–824.
29. Rickman, T., Garmany, R., Doherty, T., et al. (2001) Hypokalemia, metabolic alkalosis, and hypertension: Cushing's syndrome in a patient with metastatic prostate adenocarcinoma. *Am. J. Kidney Dis.* **37**, 838–846.
30. Miller, J.W. and Crapo, L. (1993) The medical treatment of Cushing's syndrome. *Endocr. Rev***.** **14**, 443–458.
31. Kanda, N. and Watanabe, S. (2006) Suppressive effects of antimycotics on tumor necrosis factor-alpha-induced CCL27, CCL2, and CCL5 production in human keratinocytes. *Biochem. Pharmacol*. **72**, 463–473.
32. Williams, J.G. and Maier, R.V. (1992) Ketoconazole inhibits alveolar macrophage production of inflammatory mediators involved in acute lung injury (adult respiratory distress syndrome). *Surgery* **112**, 270–277.
